# Identification of I411K, a novel missense *EYA4* mutation causing autosomal dominant non-syndromic hearing loss

**DOI:** 10.3892/ijmm.2014.1939

**Published:** 2014-09-19

**Authors:** MINXING TAN, XIAOFEI SHEN, JUN YAO, QINJUN WEI, YAJIE LU, XIN CAO, GUANGQIAN XING

**Affiliations:** 1 Department of Otolaryngology, The First Affiliated Hospital of Nanjing Medical University, Nanjing, Jiangsu 210029, P.R. China; 2 Department of Biotechnology, School of Basic Medical Sciences, Nanjing Medical University, Nanjing, Jiangsu 210029, P.R. China

**Keywords:** non-syndromic hearing loss, autosomal dominant 10, *EYA4* gene, whole-exome sequencing

## Abstract

Hearing loss is the most common sensory deficit in humans and gaining a better understanding of the underlying causes is necessary to improve counseling and rehabilitation. In the present study, a genetic analysis of a Chinese family with autosomal dominant non-syndromic progressive hearing impairment was conducted and assessed. Whole-exome sequencing in combination with a co-segregation analysis identified a novel missense mutation in *EYA4* exon 15 (c.T1301A; p.I411K). The mutation segregated with the hearing loss of the family. This mutation was not identified in the databases of 1000 Genome Project, dbSNP 130, HapMap and YH project or in matched controls. Bioinformatic analysis confirmed the pathogenic effects of this mutation. To the best of our knowledge, this is the first report to provide a description of a missense mutation in the *EYA4* gene resulting in non-syndromic hearing loss. Our results provide additional molecular and clinical information in order to gain improved understanding of the pathogenesis of *EYA4* mutations and the genotype-phenotype correlations of DFNA10 hearing loss.

## Introduction

Hearing loss is the most common sensory deficit in humans, affecting 278 million individuals worldwide and 1 in 500 newborns (1,2). Over 60% of hearing loss is caused by genetic factors, 70% of which is non-syndromic (3,4). The majority of non-syndromic hearing loss (NSHL) shows an autosomal recessive inheritance pattern (75–80%) and in 10–15% of cases the inheritance pattern is autosomal-dominant (5). At present, 54 different genetic loci for autosomal-dominant NSHL have been mapped and 28 genes have been cloned (http://hereditaryhearingloss.org).

DFNA10 is the tenth genetic locus identified for autosomal-dominant NSHL, which was mapped by linkage analysis on an American family in 1996 to chromosome 6q22.3-q23.2 (6). By further studying this American family and another unrelated Belgian family, Wayne *et al* (7) identified Eyes absent 4 (*EYA4*), a member of the vertebrate *EYA4* gene family of transcriptional activators, as the causative gene in 2001. The *EYA4* gene encodes a 640 amino acid protein, which includes a highly conserved 271 amino acid carboxy terminus termed the eya-homologous region (eyaHR) and a more divergent proline-serine-threonine (PST)-rich transactivation domain at the amino terminus (eya variable region, eyaVR) (8). The EYA proteins are components of a conserved regulatory network involved in cell-fate determination in organisms ranging from insects to humans (9). In higher animals, this network is often referred to as the Pax-Six-Eya-Dach network (PSEDN) to better reflect the vertebrate genes/proteins involved. PSEDN is both a purely transcriptional and a signal transductional network. The eyaHR and SIX family transcription factors interact to form transcriptional complexes that regulate the expression of target genes required for the development and maturation of the organ of Corti (7).

At present, six families were identified with segregation of NSHL linked to the DFNA10 locus (7,10–13). The pedigrees shared a similar phenotype: late-onset, progressive, sensorineural hearing loss (SNHL), age of onset varying from 6 to 50 years old; at onset, hearing losses were mainly situated at the midfrequencies; with increasing age, all frequencies became affected; the hearing loss was initially mild, with a spontaneous evolution to a moderate or severe hearing impairment (14). The reported *EYA4* mutations produced truncated proteins, although the eyaHR component was missing (13).

In the present study, we presented a Chinese pedigree with a novel missense *EYA4* mutation, shedding new light on the pathogenic mechanism of *EYA4* mutations.

## Materials and methods

### Family and clinical evaluation

**Figure 1:**
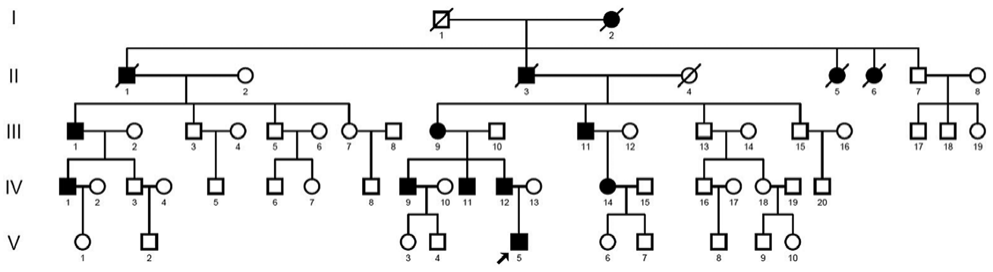
Pedigree of JSNY-023. Open symbols, unaffected; solid symbols, affected; symbols with dashed line, deceased; arrow indicates the proband.

A Chinese family classified as JSNY-023 of Han origin (Fig. 1) presented with late-onset, progressive hearing loss. Approval for the study was obtained from the Ethics Committee of the Nanjing Medical University for Human Studies. Informed consent was obtained from the participants or the parents of minors. All the individuals were evaluated through otological examination and audiological evaluations including pure-tone audiometry, immittance, auditory brainstem response (ABR), and distortion product otoacoustic emissions (DPOAEs). Medical histories, including degree of hearing loss, age of onset, progression of hearing impairment, use of aminoglycosides, noise exposure and other relevant clinical manifestations, were collected by means of a questionnaire. Information on deceased family members was obtained from relatives. High-resolution CT scan of the temporal bone, electrocardiography and echocardiography were conducted on the proband (V-5).

Peripheral blood samples were obtained from the 23 family members (9 affected and 14 unaffected). Genomic DNA was extracted using the blood genomic DNA extraction kit (DP319; Tiangen Biotech, Beijing, China) following the manufacturer’s instructions. Prior to whole-exome sequencing, the frequent deafness genes (*GJB2*, *SLC26A4*, *GJB3* and *MT-RNR1*) present in Chinese populations were excluded as the causative factor in the proband, by direct PCR-Sanger sequencing. Samples from 148 unrelated normal-hearing individuals and 53 sporadic NSHL patients were also collected and served as controls.

### Whole-exome sequencing and variation analysis

The genomic DNA of three affected individuals (IV-9, IV-12 and V-5) and one normal-hearing member (IV-13) from family JSNY-023 was enriched using the Agilent SureSelect Human All Exon kit V1.0 (Agilent Technologies Inc., Santa Clara, CA, USA). The captured DNA libraries were loaded onto the Illumina HiSeq 2000 for sequencing. Base calling was performed with Illumina base calling software V1.7 and the sequence of each subject was generated as 90-bp pair-end reads. The sequenced raw data were aligned to UCSC hg19 with SOAPaligner. Subsequently, the clean reads located in the target region were collected and the duplicate reads were filtered out. Sequencing quality was initially evaluated based on the Illumina GERALD report. The quality of the reads and the consensus sequence were calculated by SOAPsnp.

SNPs and Indels (insertions and deletions) were passed to the Genome Analysis Toolkit (GATK 1.0.4705) for identification of breakpoints. Single nucleotide variants (SNVs) and Indels were filtered against exome data from the 1000 Genome Project (1000 genomes release_20100804), dbSNP 130 (http://hgdownload.cse.ucsc.edu/goldenPath/hg18/database/snp130.txt.gz.), a HapMap (2010-08_phaseII+III) with a minor allele frequency of >0.5%, and the YH project, respectively. SNPs and Indels affecting the coding sequence were annotated using Seattle Seq annotation to predict the protein function effect of the variants.

### Sanger sequencing

Sanger sequencing was performed to determine whether any of the candidate gene variants co-segregated with the hearing loss of this family. Primers flanking the candidate loci were designed with Primer 5.0 software. As for the analysis of the suspected *EYA4* mutation, PCR primers were used: Forward, 5′-CCAAGAGTGAGG CAATGAG-3′ and reverse, 5′-TCGGTACTGTAACACC CAAA-3′. The shared variants of three affected individuals after filtering were PCR amplified and analyzed on an automated sequencer (ABI 3730 Applied Biosystems). Sequencing data were compared pair-wisely with the Human Genome database.

### Bioinformatic analysis

Three-dimensional (3D) structures for human wild-type EYA4 protein and its mutant (p.I411K) were predicted using the SWISS-MODEL (http://swissmodel.expasy.org/). Structure optimization and modifications were made with Visual Molecular Dynamics software support. Functional consequences of the protein variant were evaluated with PolyPhen2 (http://genetics.bwh.harvard.edu/pph2/) and SIFT (http://siftdna.org/www/Extended_SIFT_chr_coords_submit.html).

## Results

### Clinical findings

The pedigree of the family includes 59 members in five generations and shows autosomal-dominant inheritance. Fourteen individuals were diagnosed as having hereditary SNHL by complete audiological evaluation and medical history collection. Of nine affected individuals available for the present study, the self-reported age at onset of hearing loss ranged from 8 to 38 years. Pure tone audiograms showed that they exhibited bilaterally symmetric, mild to severe SNHL (Table I). At onset, hearing impairment was usually mild and detected at the mid-frequencies, resulting in an audiometric profile commonly referred to as a ‘cookie-bite’ pattern. Along with progression, hearing loss began to involve other frequencies. Other audiological examinations including immittance, ABR and DPOAEs in affected individuals revealed cochlear involvement. Aside from hearing loss, the patients were phenotypically normal. None of the patients complained of vestibular symptom. Imaging studies of the temporal bone and cardiac examinations of the proband were normal.

### Exome sequencing and variant analysis

Whole-exome sequencing was performed to three affected individuals and one family spouse for identification of potential variants (Table II). After mapping to the human genome reference sequence (NCBI Build 36.3, hg19), we identified on average 147,635 SNPs and 11,994 Indels in coding regions or introns. Given that these patients were related and expected to share the disease-causing variant, a total of 10,054 variants were screened among them. Of those, 69 variants, including 51 non-synonymous SNPs, splice acceptor and donor sites, and 18 Indels, were predicted to potentially have a functional impact on the gene. Through Sanger sequencing and co-segregation analysis, we eventually identified 20 variants (including 14 rare SNPs and 6 Indels) in presented sequences that co-segregated with the deafness phenotype in the 3 patients (Table III).

### Verification of EYA4 mutation

**Figure 2:**
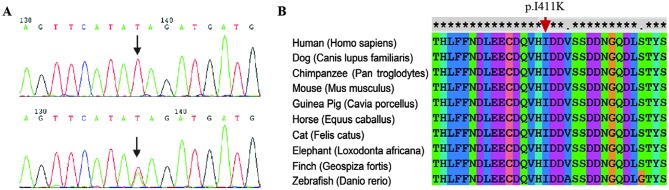
Novel p.I411K mutation identified in JSNY-023 family. (A) Partial sequence chromatograms of *EYA4* exon 15 in unaffected (upper panel) and affected (lower panel) family members. (B) Multiple amino acid sequence alignment of EYA4 protein showing high conservation of the I411 across species.

**Figure 3:**
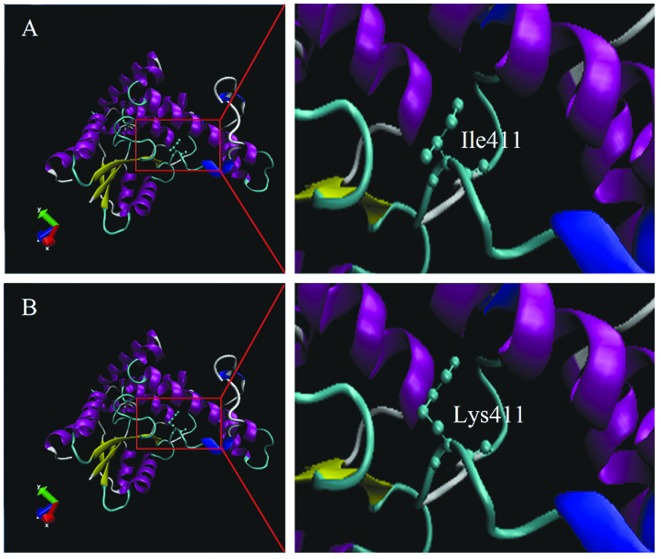
Comparison of (A) normal and (B) mutated EYA4 predicted structures.

We performed segregation analysis by Sanger sequencing on the 14 SNPs and 6 Indels, using the available 9 clinically affected subjects and 14 phenotypically normal relatives of family JSNY-023. Analyses of the *EYA4* gene identified a heterozygous T>A missense mutation (c.T1301A) in exon 15 (Fig. 2A), result in an isoleucine to lysine substitution (p.I411K). This mutation was present in the affected family members and three unaffected relatives aged <6 (V-1, V-4 and V-7), and absent in another 11 phenotypically normal relatives, 148 unrelated control subjects and 53 sporadic patients of Chinese background. Thus, the mutation c.T1301A in *EYA4* was faithfully co-segregated with the hearing loss phenotype of this family. The isoleucine residue at 411 in *EYA4* is highly conserved across human, dog, chimpanzee, mouse, guinea pig, horse, cat, elephant, finch and zebrafish (Fig. 2B). By 3D structure modeling, the mutation p.I411K was detected to change the shape of protein in this region (Fig. 3). Functional consequences of the protein variant predicted to be damaging, were evaluated with SIFT and PolyPhen2 (with a score of 0.953).

Aside from mutation c.T1301A in *EYA4* gene, none of the other 19 candidate variants was detected to be co-segregated with the hearing loss phenotype of family JSNY-023.

## Discussion

In the present study, whole-exome sequencing combined with co-segregation analysis identified a novel *EYA4* mutation, c.T1301A, in a Chinese family with autosomal-dominant NSHL, which predicts an isoleucine-to-lysine substitution (p.I411K). The following evidence suggests that the novel missense mutation is pathogenic rather than a benign polymorphism. Firstly, the variant co-segregated with the phenotype of DFNA10 in the affected family members and was not detected in the panel of 148 unrelated normal controls. Secondly, the isoleucine residue at 411 in EYA4 was highly conserved across species. The p.I411K variant is located at eyaHR, which is crucial for the function of the protein. This mutation is predicted to be damaging by both SIFT and PolyPhen2. Thirdly, according to Fleming *et al* (15), non-conservative substitutions at fixed or conservative sites and conservative substitutions at fixed sites are likely to affect function in humans.

It was believed that *EYA4* mutations led to syndromic and non-syndromic SNHL. Six human *EYA4* mutations including two non-sense mutations, three frameshift mutations and one splice site mutation have been previously reported to cause dominant NSHL (7,10–13), none of which has an associated cardiac phenotype. These mutations produced truncated proteins with a missing eyaHR component. In addition, *EYA4* mutation was also reported to cause dilated cardiomyopathy accompanying SNHL in a single large family. In this family, a 4,846-bp genomic deletion was detected that resulted in loss of the eya domain (eyaHR) as well as part of the variable region (eyaVR) (16). In the present family, the hearing loss phenotype is similar to that which has been reported for DFNA10 hearing loss, i.e., late-onset, progressive, sensorineural and non-syndromic, and the novel c.T1301A mutation creates a p.I411K substitution in the eyaHR. To the best of our knowledge, this is the first report of a missense mutation in *EYA4* leading to DFNA10 hearing loss. Our results provide support for the hypothesis that the deafness phenotype (syndromic or non-syndromic) is correlated with the *EYA4* mutation position: Mutations affecting only the eya domain cause SNHL alone, whereas mutations affecting the eya domain and variable region lead to SNHL and cardiac phenotype (12,16).

The molecular pathogenic mechanism of NSHL associated with *EYA4* mutations remains to be found, but may involve loss of gene function and haploinsufficiency through reduced gene dosage, expression or protein activity. EYA proteins interact with members of SIX and DACH protein families in a conserved network that regulates the early embryonic development and post-developmentally continued function of the mature organ of Corti (7). The haploinsufficiency of *EYA4* may lead to inadequate cochlear transcriptional regulation and function maintenance (11,13), causing SNHL, even if the mutant proteins are present in the cells and partially functional (16). In addition, members of the *eya* gene family were suggested to induce apoptosis by triggering the caspase-dependent and -independent pathways (17). Abnormal ear functions of cochlea and vestibule, have been shown in caspase-3-deficient mice (18). Thus, the apoptotic deficiency due to *EYA4* mutation may also be involved in human DFNA10 hearing loss (10). Wang *et al* (19) indicated that Eya4 regulates Na^+^/K^+^-ATPase, which is crucial for the development of mechanosensory cells of the inner ear and the maintenance of cardiac function in zebrafish, which potentially provides a mechanism by which human *EYA4* mutations cause hearing loss and heart disease.

Makishima *et al* (12) have found that several genetic or environmental factors may modify the DFNA10 phenotype. In family JSNY-023, the reported age of onset was 8 years in the proband, which was 10 years earlier than the second early age of onset (Table I). Further insights showed that other affected individuals from the same branch (III-9, IV-9, IV-11 and IV-12) shared an earlier onset age than those members from the extended family (III-1, III-11, IV-1 and IV-14). This phenomenon suggests that except for *EYA4*, other genes potentially contribute to the phenotype. There were three phenotypically normal individuals (V-1, V-4 and V-7), aged <6 in this family who tested positive for the *EYA4* mutation. Their onset of hearing loss is anticipated during follow-up, which may support the finding.

In conclusion, we have identified a novel DFNA10 mutation, p.I411K, in a Chinese family. To the best of our knowledge, it is the first description of a missense mutation in *EYA4* gene leading to NSHL. The mutation affects one single amino acid in the eya domain which is highly conserved across species. Our results provide additional molecular and clinical information to gain a better understanding of the pathogenesis of *EYA4* mutations and the genotype-phenotype correlations of DFNA10 hearing loss.

## Figures and Tables

**Table I:** Summary of clinical data for affected individuals of family JSNY-023.

		Age (years) (dBHL)		Pure-tone average^a^			
					
Subjects	Gender	At testing	At onset	Left	Right	Audiogram shape	Degree of hearing loss
III-1	Male	51	38	41.25	45	Flat-sloping	Moderate
III-9	Female	64	20	83.75	71.25	Flat	Severe
III-11	Male	57	30	56.25	58.75	Flat-sloping	Moderate-severe
IV-1	Male	31	31	27.5	28.75	Cookie-bite	Mild
IV-9	Male	39	18	60	62.5	Flat-sloping	Moderate-severe
IV-11	Male	35	18	66.5	63.75	Flat-sloping	Moderate-severe
IV-12	Male	35	20	61.25	61.25	Flat-sloping	Moderate-severe
IV-14	Female	33	32	31.25	33.75	Cookie-bite	Mild
V-5	Male	10	8	40	38.75	Cookie-bite	Mild

a Pure-tone average was calculated by hearing threshold levels in speech frequencies 0.5, 1, 2 and 4 kHz.

**Table II:** Summary of exome sequencing data for each sample.

	Subjects			
	
Exome capture statistics	IV-13	IV-9	IV-12	V-5
Total effective data yield (Mb)	6619.47	8094.35	8093.93	8104.01
Total effective reads	66194702	80943452	80939348	81040066
Uniquely mapping reads rate	84.19%	84.10%	83.90%	83.87%
No-mismatch mapping reads rate	71.79%	72.18%	70.18%	71.05%
Mismatch alignment bases rate	0.49%	0.47%	0.52%	0.51%
The ratio of reads alignment to reference genome	98.49%	98.25%	98.11%	98.51%
Capture efficiency rate on target regions	62.57%	62.25%	56.66%	62.04%
Capture efficiency rate on or near ±150 target regions	68.17%	66.68%	61.66%	67.64%
Capture efficiency rate on or near ±500 target regions	69.00%	67.55%	62.76%	68.49%
Mean coverage sequencing depth on official target	55	67	61	67
Fraction of official target covered	95.67%	95.57%	96.57%	96.16%
Fraction of official target covered with at least 4X	93.30%	93.24%	93.81%	94.00%
Fraction of official target covered with at least 10X	89.93%	90.31%	90.76%	91.22%
Fraction of official target covered with at least 20X	83.24%	84.59%	84.93%	86.10%

**Table III:** Candidate variants shared by three affected individuals.

No.	Chromosome	Position	Reference	Change	Gene	Substitution
1	chr3	108220603	C	T	*MYH15*	V119M
*2*	*chr6*	*109763791*	*C*	*T*	*SMPD2*	*R152C*
**3**	**chr6**	**133833878**	**T**	**A**	***EYA4***	**I411K**
4	chr7	127014845	G	A	*ZNF800*	P182L
5	chr7	131195709	G	A	*PODXL*	T195M
6	chr8	17409321	C	T	*SLC7A2*	T294M
7	chr12	52714915	C	T	*KRT83*	G69S
8	chr13	88329945	C	G	*SLITRK5*	R768G
9	chr14	92482108	C	T	*TRIP11*	R252Q
10	chr14	105416749	G	A	*AHNAK2*	S1680L
11	chr16	4848143	C	T	*ROGDI*	V192I
12	chr16	88498341	C	T	*ZNF469*	P1460L
13	chr19	11942931	T	C	*ZNF440*	C314R
14	chr21	47754587	C	T	*PCNT*	R182C
15	chr3	111261145	-	+T	*CD96*	Insertion
16	chr3	156527065	T	-	*PA2G4P4*	Deletion
17	chr5	111496904	C	-	*EPB41L4A-AS1*	Deletion
18	chr11	57995879	G	-	*OR10Q1*	Deletion
19	chr11	61511925	-	+G	*DAGLA*	Insertion
20	chr14	92471390	-	+T	*TRIP11*	Insertion

Bold denotes the *EYA4* I411K mutation.
